# Nanog overexpression enhances the therapeutic efficacy of ADMSCs in AMI rats via the upregulation of JAK/STAT3 signaling and cyclin-mitochondrial expression

**DOI:** 10.7150/ijbs.112824

**Published:** 2025-07-04

**Authors:** Hsu-Ting Yen, David Kwan Ru Huang, Xian-Wu Lan, Jui-Ning Yeh, Yi-Ling Chen, Chi-Ruei Huang, Yi-Ting Wang, Hon-Kan Yip, Pei-Hsun Sung, Sheung-Fat Ko

**Affiliations:** 1 Division of Thoracic and Cardiovascular Surgery, Department of Surgery, Kaohsiung Chang Gung Memorial Hospital and Chang Gung University College of Medicine, Kaohsiung 833401, Taiwan.; 2 Kaohsiung Municipal Fong-Shan Hospital, Kaohsiung 830025, Taiwan.; 3 Department of Cardiology, the First Hospital of Jinan University, GuangZhou 510630, China.; 4 Institute of Nephrology and Blood Purification, the First Affiliated Hospital of Jinan University, Jinan University, Guangzhou 510632, China.; 5 Division of Cardiology, Department of Internal Medicine, Kaohsiung Chang Gung Memorial Hospital and Chang Gung University College of Medicine, Kaohsiung 833401, Taiwan.; 6 Institute for Translational Research in Biomedicine, Kaohsiung Chang Gung Memorial Hospital, Kaohsiung 833401, Taiwan.; 7 Center for Shockwave Medicine and Tissue Engineering, Kaohsiung Chang Gung Memorial Hospital, Kaohsiung 833401, Taiwan.; 8 Department of Medical Research, China Medical University Hospital, China Medical University, Taichung 404333, Taiwan.; 9 School of Medicine, College of Medicine, Chang Gung University, Taoyuan 333323, Taiwan.; 10 Department of Radiology, Kaohsiung Chang Gung Memorial Hospital and Chang Gung University College of Medicine, Kaohsiung 833401, Taiwan.

**Keywords:** acute myocardial infarction, Nanog gene overexpression in adipose-derived mesenchymal stem cells, cell stress signaling

## Abstract

**Background:** This study investigated whether Nanog-overexpressing adipose-derived mesenchymal stem cells (Nanog^OE^-ADMSCs) are superior to unmodified ADMSCs in improving the left ventricular ejection fraction (LEVF) in acute myocardial infarction (AMI) patients.

**Methods:** We utilized silencing and overexpression of Nanog gene in ADMSCs and performed a wound healing assay/transwell migration assay/MTT cell viability assay/left coronary artery ligation for AMI induction. Additionally, we categorized the cells into three classes [i.e., (ADMSCs and Nanog^OE^-ADMSCs); A_1_ (ADMSCs)/A_2_ (ADMSCs + CoCl_2_)/A_3_ (Nanog^OE^-ADMSCs + CoCl_2_)/A_4_ (siRNA-Nanog-ADMSCs) + CoCl_2_); B_1_ (ADMSCs)/B_2_ (ADMSCs + H_2_O_2_)/B_3_ (Nanog^OE^-ADMSCs + H_2_O_2_)/B_4_ (siRNA-Nanog gene in ADMSCs + H_2_O_2_)], and the rats (n=50) were evenly divided into Groups 1 (sham-operated control)/2 (AMI)/3 (AMI+ADMSCs)/4 (AMI+Nanog^OE^-ADMSCs)/5 (AMI+siRNA-Nanog-ADMSCs). The hearts were harvested on Day 35.

**Results:** *In vitro* experiments revealed significantly higher ATP, relative mitochondrial DNA/Nonog gene expression, mitochondrial cytochrome C+ cell, angiogenesis and exosome-specific marker (Alix/CD81/CD63/CD9) levels in Nanog^OE^-ADMSCs than in ADMSCs. The cell viability, wound healing, and migration were highest in A1, lowest in A4, and significantly greater in A3 than in A2, whereas early/late apoptosis and intracellular and mitochondrial ROS displayed the opposite pattern of cell viability among the groups (all *P<*0.001). Additionally, the proteins expressions of phosphorylation (p) of the PI3K/Akt/mTOR, p-JAK2/p-STAT3, and Ras/Raf/MEK_1/2_/ERK_1/2_ signaling pathways were highest in A3, lowest in A4 and significantly greater in A1 than in A2 (all *P<*0.001). The levels of cell cycle proteins and mitochondrial electron transport train (ETC) complex I/II/III/IV components exhibited identical patterns as PI3K/Akt/mTOR among the groups B1 to B4 (all *P<*0.001). On Day 35, the LVEF was highest in Group 1, lowest in Group 2, significantly greater in Group 4 than in Groups 3 and 5, and significantly greater in Group 3 than in Group 5, with the opposite pattern for the LV remodeling index, infarct and fibrosis areas, and LV chamber size (all *P <* 0.0001). The p-AK/p-STAT3, p-PI3K/p-Akt/p-mTOR, and Ras/Raf/MEK_1/2_/ERK_1/2_ protein levels displayed the same pattern as the LVEF among the groups (all *P <* 0.001).

**Conclusion:** Nanog^OE^-ADMSCs rescued LVEF by upregulating JAK/STAT3-mediated cell proliferation/cell stress pathways and accelerating the cell cycle.

## Introduction

The short-term and long-term mortality of acute myocardial infarction (AMI) is linearly correlated with the infarct area and myocardial loss 1. For patients suffering from heart failure (HF) with a left ventricular ejection fraction (LVEF) <40% following AMI, the estimated 30-day mortality is greater than 40% 1. Furthermore, mortality is even higher than 70% for victims who develop cardiogenic shock in the context of AMI, regardless of the degree of hemodynamic support 1. A core concept in the survival chain of AMI is “time is muscle” 2, meaning that survival following AMI depends on how quickly the patient arrives to the emergency department and how much myocardium is preserved by reperfusion therapy 3. Nevertheless, despite the widely-used catheterization management methods and antithrombotic and cardioprotective treatments, effective therapies aimed at the recovery of damaged cardiomyocytes are lacking 4.

Increasing evidence has shown that mesenchymal stem cells (MSCs) have anti-inflammatory, immunomodulatory, and regenerative functions 5. MSCs also undergo angiogenesis and neovascularization after they differentiate into endothelial lineage cells 6, resulting in the restoration of blood flow in the ischemic zone, improvement of left ventricular (LV) function, alleviation of LV remodeling, and a reduction in recurrent hospitalization for heart failure 7. However, the occurrence of myocardial necrosis in the occluded vascular territory accompanied by excessive hypoxia and oxidative stress consistently leads to detrimental effects on the age and senescence of MSCs, consequently resulting in dysfunction of the implanted or infused stem cells 8. Furthermore, increased reactive oxygen species (ROS) inhibit MSCs proliferation, increase senescence, reduce osteogenic differentiation, and inhibit immunomodulatory function 9. These conflicting effects could partially explain why the results from blinded clinical trials of cell therapy for AMI are inconsistent and why the relevant benefits are inconclusive 10. Thus, identifying a method to increase implanted cell function to salvage the injured myocardium is highly important.

Our previous experimental studies demonstrated that the utilization of cell therapy with epigenetic modification effectively protects vital organs against functional/structural damage from AMI, dilated cardiomyopathy and acute kidney ischemia‒reperfusion injury 11-13. The therapeutic mechanisms involved in organ protection 11-13 include immunomodulation, reducing oxidative stress, alleviating DNA and mitochondrial damage, inhibiting upstream and downstream inflammatory signaling pathways, decreasing apoptosis and fibrosis, preserving organ architecture, and improving organ functions. On the basis of the results of these experiments in epigenetically and genetically modified cytotherapy, we hypothesized that cell may be possible to increase the efficacy of stem cell therapy in the context of myocardial infarction.

Nanog is a primitive/pluripotent stem cell marker and is considered a crucial regeneration factor during tissue repair 14. After AMI, Nanog is positively expressed at both the mRNA and protein levels in myocardial cells, fibroblasts and small round cells in different myocardial zones at different stages 14. All cardiomyocytes, fibroblasts and small round cells are involved in myocardial reconstruction following AMI. Nanog-positive cardiomyocytes may be responsible for early myocardial repair 14. Similarly, Nanog-positive fibroblasts and small round cells are the main sources for myocardial reconstruction. Taken together, Nanog expression in heart tissues following AMI demonstrates spatiotemporal and dynamic changes in tissue repair and cardiac regeneration, especially in the infarct zone 15. Accordingly, we have sufficient evidence to believe that Nanog overexpression in MSCs used to treat the infarcted/peri-infarcted myocardium not only enhances implanted cell function but also accelerates cardiac regeneration in the context of AMI. On the basis of these findings, Nanog gene overexpression in adipose-derived mesenchymal stem cells (Nanog^OE^-ADMSCs therapy may be better than ADMSCs therapy alone for improving heart function in rodents after AMI.

## Materials and Methods

### Ethical approval

All animal procedures were approved by the Institute of Animal Care and Use Committee at Kaohsiung Chang Gung Memorial Hospital (Affidavit of Approval of Animal Use Protocol No. 2022090101) and performed in accordance with the Guide for the Care and Use of Laboratory Animals. This work has been reported in accordance with the ARRIVE guidelines (Animals in Research: Reporting *In vivo* Experiments) 16.

The animals were housed in an Association for Assessment and Accreditation of Laboratory Animal Care International (AAALAC; Frederick, MD, USA)-approved animal facility in our hospital with a controlled temperature and light cycle (24 °C and 12/12 light cycle).

### Isolation of allogenous ADMSCs

To obtain adequate numbers of ADMSCs for individual studies, an additional twelve SD rats were utilized. Briefly, adipose tissues around the epididymis and abdomen were harvested meticulously as we have previously described 17, 18. After 14 days of culture, approximately 2.5-3.5 × 10^6^ ADMSCs were obtained from each rat.

### Hypoxia induction by cobalt chloride (CoCl_2_)

The procedure and protocol used to induce hypoxia were described in our previous report 19, and CoCl_2_ was obtained from Sigma‒Aldrich. Briefly, ADMSCs treated with or without CoCl_2_ (0 or 200 μM) as a hypoxic stimulus for 48 h in DMEM were collected for experiments.

### Transfection of ADMSCs with plasmids for Nanog gene overexpression

The procedure and protocol for this transfection have been previously described in our recent study 20. Briefly, the *NANOG* expression vector (pcDNA3.1-*NANOG*) was purchased from Genomics, Inc. (New Taipei City, Taiwan). Plasmid transfection was performed using Lipofectamine 3000 according to the manufacturer's instructions. First, 15 µg of the Nanog expression vector and 30 µl of Lipofectamine 3000 were incubated at room temperature for 15 minutes, followed by overnight incubation at 37 °C in a humidified atmosphere of 5% CO_2_ and Lipofectamine. Additional experiments were subsequently performed.

### Procedure and protocol for siRNA *Nanog* gene-transfected ADMSCs

siRNA-*Nanog* was purchased from Ambion, Inc. The siRNA 1 sequences were as follows: sense, 5'-CCCUGAUUCUUCUAGCAAUTT-3', and antisense, 5'-AUUGCUAGAAGAAUCAGGGCT-3'. The siRNA 2 sequences were as follows: sense, 5'-GAACGCGUCUGGAAACCUUTT-3', and antisense, 5'-AAGGUUUCCAGACGCGUUCAT-3'. Transient transfection of cells with the siRNA mixture (siRNA1+siRNA2) was conducted with Lipofectamine™ RNAiMAX Transfection Reagent (Invitrogen, Life Technologies, Carlsbad, CA, USA) according to the manufacturer's instructions with slight modifications. The cells were replated 24 h before transfection at a density of 1 × 10^6^ cells in 7 ml of fresh culture medium in a 10-cm plastic dish. The next day, the cells were incubated with 100 pmol of the indicated siRNA at room temperature for 15 minutes with Lipofectamine™ RNAiMAX Transfection Reagent. The cells were incubated with the siRNA/liposome complex at 37 °C in a humidified atmosphere of 5% CO_2_ before being harvested. Western blotting and RT‒PCR were performed to verify the efficiency of gene silencing.

### MTT cell viability assay

The procedure and protocol for this assay have been described in our previous report 21. Briefly, cell proliferation/growth was determined by the MTT assay. Approximately 2 × 10^3^ cells in 100 µL of medium were seeded into the wells of a 96-well plate and incubated for the indicated duration. At the end of the incubation, MTT solution was added to each well, and the purple crystalline sediment was dissolved in DMSO. The absorbance was read at 540 nm using an ELISA reader. The absorbance value was used to represent the cell number indicating cell viability.

### Wound healing assay

The procedure and protocol for this assay have been described in our previous report 21. Briefly, for the wound healing assay, 3.5 × 10^4^ cells were seeded in 12 wells with linear spacer inserts. Following overnight cell culture, a regular and defined “wound” within the cell monolayer was created by removing the linear spacer inserts. After washing with phosphate-buffered saline, the wells were either left untreated or treated with or without Mel (100.0 μM) or cisplatin (6.0 µM). After a 24-h incubation, the migration of the cells into the denuded areas in the marked region was monitored. Migration was captured with microphotography, and the total migration distance was determined using ImageJ software (National Institutes of Health, Bethesda, MD, USA).

### Transwell migration assay for assessment of ADMSCs migratory capacity

The procedure and protocol for this assay have been described in our previous report 21. Briefly, the cells were first trypsinized, and then 3.5 × 10^4^ cells were added to the Boyden chambers (8 μm pore size; *Millipore, Billerica*, MA, USA) with 0.5% FBS-containing medium, and assay media containing 10% FBS was added to the culture plates. After 24 h of incubation, the nonmotile ADMSCs/Nanog^OE^-ADMSCs located at the top of the filter were removed, and the motile cells at the bottom of the filter were fixed with methanol and stained with a one-tenth dilution of Giemsa (Sigma Corporation, Cream Ridge, NJ, USA). The number of migrated cells in each chamber was carefully counted in five randomly chosen fields under a microscope for three independent experiments.

### Animal model of acute myocardial infarction (AMI)

The AMI induction protocol was performed as described in our previous studies 22, 23. In brief, all the rats were anesthetized, and the heart of each rat was exposed via a left thoracotomy. Sham-operated rats (SCs) underwent thoracotomy only, whereas AMI was induced in the other groups via left coronary artery (LCA) ligation. After the procedure, the thoracotomy wound was closed, and all the rats recovered from anesthesia.

The animals (n = 50) were equally categorized into Group 1 [sham-operated control (SC)], Group 2 [AMI (i.e., induction by left coronary artery (LCA) ligation], Group 3 [AMI + allogenic ADMSCs (6.0 × 10^5^ cells intramyocardial injection + 1.2 × 10^6^ cells by intravenous administration 3 h after AMI induction)], Group 4 [AMI + Nanog^OE^-ADMSCs (6.0 × 10^5^ cells intramyocardial injection + 1.2 × 10^6^ cells by intravenous administration 3 h after AMI induction)] and Group 5 [AMI + siRNA-Nanog-ADMSCs (6.0 × 10^5^ cells intramyocardial injection + 1.2 × 10^6^ cells by intravenous administration 3 h after AMI induction)]. The dose and the route of administration of ADMSCs in the present study were based on our previous report with some modification 24.

### Euthanasia of animals

On Day 35 after AMI induction, the animals in each group were euthanized. Specifically, the animals were euthanized under anesthesia with 2.0% isoflurane inhalation at a steady respiratory rate, after which more than 10 ml of blood was exsanguinated by a needle puncture into the LV chamber to draw the cardiac blood to achieve euthanasia. This procedure usually requires five minutes (i.e., 2.0% isoflurane inhalation was maintained for five minutes). The heart of each animal was harvested after respiratory arrest for use in subsequent experiments.

### Functional assessment by echocardiography

The protocol for transthoracic echocardiography was conducted on the basis of our previous studies 22, 23. In each experimental group, transthoracic echocardiography was performed using a Vevo 2100 (Visualsonics, Toronto, Ontario, Canada) prior to AMI induction and at Days 14 and 35 after AMI induction. M-mode standard two-dimensional (2D) left parasternal long-axis echocardiographic examination was conducted. Left ventricular internal dimensions, including the left ventricular end-systolic diameter (LVESd) and left ventricular end-diastolic diameter (LVEDd), were measured at the mitral valve and papillary levels of the left ventricle during at least three consecutive cardiac cycles. The left ventricular ejection fraction (LVEF) was evaluated as follows: LVEF (%) = [(LVEDd3-LVESd3)/LVEDd3] × 100%. Transthoracic echography was performed on Day 0 prior to and on Days 14 and 35 after AMI induction before the animals were euthanized.

### Western blot analysis

The protocol for this analysis has been described in our previous and recent reports 18, 20-22. Briefly, equal amounts (30 µg) of protein extracts from heart tissues were separated via 8-12% SDS‒PAGE. After electrophoresis, the separated proteins were transferred onto a polyvinylidene difluoride (PVDF) membrane (Amersham Biosciences, Amersham, UK). Nonspecific sites were blocked by incubation of the membrane in blocking buffer [5% nonfat dry milk in T-TBS (TBS containing 0.05% Tween 20)] at room temperature for one hour. The membranes were subsequently incubated with the indicated primary antibodies [phosphorylated (p)-Akt (1:1,000, Cell Signaling), Akt (1:1,000, Cell Signaling), p-mTOR (1:3,000, Abcam), mTOR (1:3,000, Abcam), p-PI3K (1:1,000, Cell Signaling), PI3K (1:1,000, Cell Signaling), NOX1 (1:1,000, Sigma), NOX2 (1:1,000, Sigma), p-JAK2 (1:3,000, Abcam), p-STAT3 (1:3,000, Abcam), cleaved caspase3 (1:1,000, Cell Signaling), cleaved caspase 9 (1:1,000, Abcam), RAS (1:1,000, Cell Signaling), RAF (1:1,000, Cell Signaling), p-MEK (1:1,000, Cell Signaling), p-ERK (1:3,000 Millipore), OXPHOX (mitochondria complexes) (1:5,000, Abcam), cyclin D (1:3,000, Abcam), Cyclin E (1:3,000, Abcam), CDK2 (1:3,000, Abcam), CDK4 (1:3,000, Abcam), Alix (1:3,000, Abcam), CD81 (1:3,000, Arrigo), CD63 (1:3,000, Arrigo), CD9 (1:3,000, Arrigo), Nanog (1:1,000, Abcam), and actin (1:10,000, Merck)] for 1 hour at room temperature. Horseradish peroxidase-conjugated anti-rabbit or anti-mouse immunoglobulin IgG (1:2,000, Cell Signaling, Danvers, MA, USA) was used as a secondary antibody for one hour of incubation at room temperature. After being washed, the immunoreactive membranes were visualized using enhanced chemiluminescence (ECL; Amersham Biosciences, Amersham, UK) and exposed to medical X-ray film (FUJI).

### Immunohistochemical (IHC) staining

This staining procedure has been described in our recent reports 18, 20-22. Briefly, for IHC staining, rehydrated paraffin sections were first treated with 3% H_2_O_2_ for 30 minutes and then incubated with Immuno-Block reagent (BioSB, Santa Barbara, CA, USA) for 30 minutes at room temperature. Five sections of heart specimens from each rat were analyzed. For quantification, three randomly chosen HPFs (400x for the IHC study) were analyzed in each section.

### Flow cytometric analysis for detecting apoptosis and ROS levels in cultured ADMSCs

The percentages of viable and apoptotic cells were determined using flow cytometry (Beckman Coulter Life Sciences) with double staining with annexin V and propidium iodide (PI). The early phase of apoptosis was defined as annexin V+/PI-, whereas the late phase of apoptosis was defined as annexin V+/PI+. Flow cytometric analyses for intracellular and mitochondrial reactive oxygen species (ROS) were conducted for total cellular ROS (i.e., by H_2_DCFDA assay) and mitochondrial ROS (i.e., by MitoSOX assay), respectively.

### Statistical analysis

The quantitative data are expressed as the means ± SDs. Statistical analysis was performed by one-way ANOVA followed by the Bonferroni multiple comparison post hoc test. SAS statistical software for Windows version 8.2 (SAS Institute, Cary, NC, USA) was utilized. A probability value < 0.05 was considered statistically significant.

## Results

### Nanog^OE^-ADMSCs promote ADMSCs proliferation in a hypoxic environment (Figure 1)

To address the ability of Nanog gene overexpression in ADMSCs (Nanog^OE^-ADMSCs) to protect ADMSCs against hypoxia-induced damage, the cells were categorized as follows: A_1_ (ADMSCs only), A_2_ (ADMSCs + CoCl_2_), A_3_ (Nanog^OE^-ADMSCs + CoCl_2_) and A_4_ (silencing the Nanog gene in ADMSCs (i.e., siRNA-Nanog-ADMSCs) + CoCl_2_]). The cells were collected after different time points of cell culture for analysis. The results demonstrated that the cell viability at 24, 48 and 72 h was highest in A_1_, lowest in A4, and significantly greater in A_3_ than in A_2_. Additionally, the migratory assay demonstrated that the cell migratory ability displayed an identical pattern as the cell viability among the groups. Furthermore, the wound healing ability also displayed a pattern identical to that of cell viability among the groups. Our findings indicate that Nanog^OE^-ADMSCs were more favorable than ADMSCs for cell survival and proliferation under hypoxic conditions.

### Nanog^OE^ in ADMSCs suppresses cell apoptosis and ROS production in a hypoxic environment (Figure 2)

**Figure 1:**
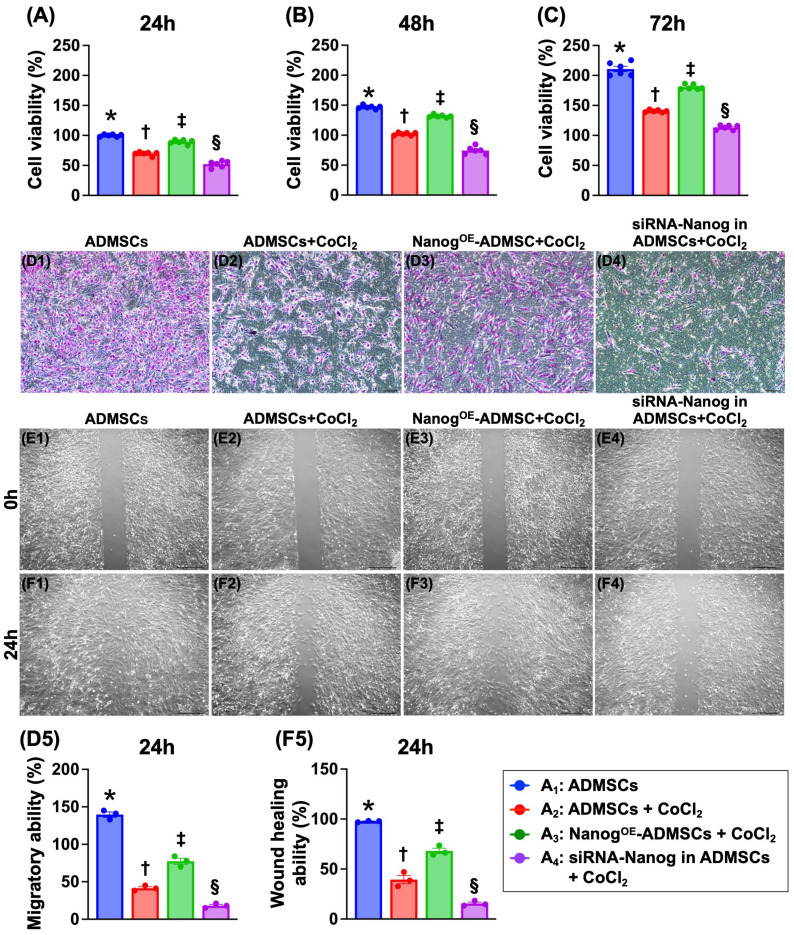
**Nanog^OE^-ADMSCs ensured the AMDMSCs survival in hypoxic environment. A)** MTT assay for identification of cell viability at 24 h, * vs. other groups with different symbols (†, ‡, §), *P<*0.0001. **B)** MTT assay for identification of cell viability at 48 h, * vs. other groups with different symbols (†, ‡, §), *P<*0.0001. **C)** MTT assay for identification of cell viability at 48 h, * vs. other groups with different symbols (†, ‡, §), *P<*0.0001. **D1 to D4)** Illustrating the microscopic finding (100x) for identification of cell migratory ability (pink color). Scale bar in right lower corner represents 50µm. **D5)** Analytical result of cell migratory ability, * vs. other groups with different symbols (†, ‡, §), *P<*0.0001. **E1 to E4)** Illustrating the wound healing process at baseline. Analytical result, *P*>0.5. **F1 to F4)** Illustrating the wound healing process at 24 h. **F5)** Analytical result of wound healing ability, * vs. other groups with different symbols (†, ‡, §), *P<*0.0001. Note: * indicated the control group. All statistical analyses were performed by one-way ANOVA, followed by Bonferroni multiple comparison post hoc test (n=3-6) for each group). Symbols (*, †, ‡, §) indicate significance (at 0.05 level). Cell grouping: A_1_ = ADMSCs, A_2_ = ADMSCs + CoCl_2_, A_3_ = Nanog^OE^-ADMSCs + CoCl_2_, A4 = siRNA-Nanog in ADMSCs + CoCl_2_. ADMSCs = adipose-derived mesenchymal stem cells; Nanog^OE^ = overexpression of Nanog gene. CoCl_2_ serviced as a hypoxia condition.

To address whether Nanog^OE^-ADMSCs are better able to mitigate the effects of hypoxia than ADMSCs, ADMSCs and Nanog^OE^-ADMSCs were utilized and categorized into A_1_ to A_4_, as shown in Figure 1. Flow cytometric analysis revealed that early and late apoptosis and intracellular and mitochondrial ROS levels were highest in A_4_, lowest in A_1_ and significantly lower in A_3_ than in A_2_. These findings demonstrate that Nanog^OE^-ADMSCs ensure cell survival in hypoxic environments.

Nanog protein levels were highest in A_3_, lowest in A_4_ and significantly higher in A_1_ than in A_2_, indicating that the gene modulation procedure was successful in our *in vitro* study.

### ATP concentration, mitochondrial DNA expression, mitochondrial cytochrome-C and angiogenesis are increased by Nanog gene overexpression (Figure 3)

To evaluate whether Nanog gene overexpression in ADMSCs enhances cellular biological activity and mitochondrial biogenesis, the cells were classified as ADMSCs only or Nanog^OE^-ADMSCs. Compared with those in the ADMSCs group, Nanog gene and protein expression and ATP concentrations significantly increased in the Nanog^OE^-ADMSCs group. Additionally, relative mitochondrial DNA expression and the number of mitochondrial cytochrome C+ cells were significantly greater in Nanog^OE^-ADMSCs than in ADMSCs. Moreover, a Matrigel assay demonstrated that angiogenesis was significantly greater in rat circulatory-derived endothelial progenitor cells (EPCs) treated with Nanog^OE^-ADMSCs-derived conditioned medium (i.e., supernatant) than in EPCs treated with ADMSCs-derived conditioned medium. The culture medium was also collected for isolation of exosomes, followed by Western blot analysis. We found that typical exosome biological markers, including Alix, CD81, CD63 and CD9, were significantly more abundant in Nanog^OE^-ADMSCs than in ADMSCs, indicating that exosomes might, at least in part, participate in enhancing EPC angiogenesis. Accordingly, our findings indicate that Nanog gene manipulation in ADMSCs was successful, resulting in increased cellular biological activity and increased mitochondrial biogenesis and ATP energy.

### Nanog gene overexpression in ADMSCs suppresses the levels of proteins related to oxidative stress and cellular apoptosis and upregulates the expression of proteins related to cell stress signaling (Figure 4)

To investigate the impact of Nanog gene overexpression on the regulation of cell stress/proliferation signaling and oxidative stress/apoptosis, the cell groups were categorized as described in Figure 1 (A_1_ to A_4_). The protein expression levels of NOX-1 and NOX-2, two indices of oxidative stress, and the protein expression levels of cleaved caspase 3 and cleaved caspase 9 were significantly lower in A_1_ and A_3_ than in A_2_ and A_4_ and significantly greater than those in A4 and A_2_. However, these levels did not differ between A_1_ and A_3_.

It is well known that the tyrosine-phosphorylated JAK/STAT signaling pathway mediates downstream events, including cell stress signals that are transported into the nucleus through the nuclear membrane to regulate specific genes. Our *in vitro* results revealed that the protein expression levels of phosphorylated JAK/STAT3 and its downstream signaling proteins, including p-PI3K, p-Akt, p-m-TOR (cell proliferation signaling), and Ras, Raf, MEK1/2 and ERK1/2 (cell stress signaling), were significantly lower in A_4_, highest in A_3_ and significantly lower in A_1_ than in A_2_. These findings suggest that Nanog gene overexpression promotes cell proliferation and cell stress signaling.

### Nanog gene expression activates the cell cycle and upregulates the mitochondrial electron transport chain (ETC) under oxidative stress conditions (Figure 5)

To verify whether Nanog gene expression activates ETC complexes I-IV and accelerates the cell cycle, the cells were categorized into B_1_ (ADMSCs only), B_2_ (ADMSCs + H_2_O_2_ (150 μM)), B_3_ (Nanog^OE^-ADMSCs + H_2_O_2_ (150 μM)), and B_4_ (siRNA-Nanog gene in ADMSCs + H_2_O_2_ (150 μM)). Western blot analysis revealed that the protein expression levels of Cyclin D, Cyclin E, CKD2 and CKD4, which are four indices of cell cycle biomarkers, and the protein expression levels of ETC complexes I to IV were lowest in A_4_, highest in A_3_ and significantly greater in A_1_ than in A_2_.

### Time courses of the LVEF and left ventricular remodeling index (LVRI) (Figure 6)

At baseline, the LVEF, LVRI and LVEDd did not differ among the groups. However, at 14 days after AMI induction, the LVEF was highest in Group 1 (i.e., SC), lowest in Groups 2 (AMI only) and 5 (AMI + siRNA-Nanog in ADMSCs) and significantly greater in Group 4 (AMI + Nanog^OE^-ADMSCs) than in Group 3 (AMI + ADMSCs). On the other hand, the LVRI (i.e., the ratio between the left ventricular mass and end-diastolic volume) and LVEDd, two indices of LV remodeling, exhibited the opposite pattern of LVEF among the groups.

Additionally, on Day 35 after AMI induction, the LVEF remained highest in Group 1, lowest in Group 2, significantly lower in Group 5 than in Groups 3 and 4, and significantly greater in Group 4 than in Group 3. On the other hand, the LVRI and LVEDd displayed the opposite pattern of LVEF in this experiment. Our findings indicate that Nanog^OE^-ADMSCs treatment was superior to ADMSCs treatment in preserving LV function and attenuating LV remodeling.

### Levels of proteins related to cell proliferation and cell-stress signaling are altered in the LV myocardium on Day 35 after AMI induction (Figure 7)

The protein expression levels of p-JAK and p-STAT3, members of a common pathway for the transduction of signals from the extracellular level to the intracellular environment, were significantly lower in Group 2 than in Group 1, and these proteins were significantly upregulated in Group 3 and further significantly upregulated in Group 4. However, these parameters were significantly lower in Group 5 than in Groups 3 and 4.

Additionally, the protein expression levels of p-PI3K, p-Akt and p-mTOR, which are three indicators of cell proliferation signaling, and the protein expression levels of RAS, RAF, p-MEK1/2 and ERK1/2, which are four biomarkers of cell stress signaling, displayed patterns similar to those of p-JAK/p-STAT3 signaling proteins among the groups.

### Protein expression of the cell cycle and mitochondrial ETC complexes is altered in the LV myocardium by Day 35 after AMI induction (Figure 8)

The protein expression levels of cyclin D, cyclin E, CDK2 and CDK4, which are four indices of cell cycle biomarkers, were significantly lower in Group 2 than in Group 1. In addition, the expression levels of these proteins were significantly greater in Group 3 and significantly greater in Group 4 than in Group 1. However, these parameters were significantly lower in Group 5 than in Groups 3 and 4. Additionally, the protein expression levels of mitochondrial ETC complexes I to IV also displayed a pattern identical to that of the cell cycle proteins among the groups.

### Protein expression is associated with oxidative stress and apoptosis, the LV myocardial infarct area and cardiomyocyte size (Figure 9)

The protein expression levels of NOX-1 and NOX-2, which are two indicators of oxidative stress, and the protein expression levels of cleaved caspase 3 and cleaved caspase 9, which are two indicators of apoptosis, were significantly greater in Group 2 than in Group 1 but were significantly lower in Group 3 and significantly lower in Group 4. However, these parameters were significantly reversed in Group 5 compared with Groups 3 and 4. H&E staining revealed that the LV infarct area was lowest in Group 1, highest in Group 2, significantly lower in Group 4 than in Groups 3 and 5 and significantly lower in Group 3 than in Group 5. Additionally, the cardiomyocyte size of the LV myocardium exhibited an identical pattern of infarct area among the groups.

## Discussion

**Figure 10:**
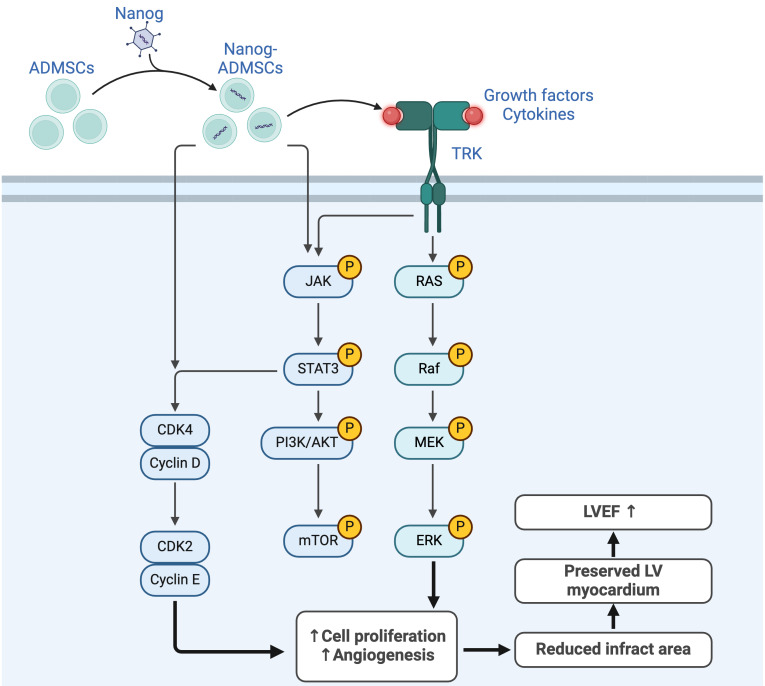
** Underlying mechanism of Nanog gene on affecting the outcomes of AMI.** Figure 10 illustrates the overexpression of Nanog gene on ADMSCs (i.e., Nanog^OE^-ADMSCs) on upregulating the cell stress/cell proliferation signalings, resulting on improving the outcomes in setting of AMI. ADMSCs = adipose-derived mesenchymal stem cells; AMI = acute myocardial infarction; LVEF = left ventricular ejection fraction.

This study investigated the therapeutic impact of Nanog^OE^-ADMSCs on preserving heart function and suppressing molecular‒cellular perturbations, which has several notable implications. First, the *in vitro* study results demonstrated that Nanog gene overexpression enhanced cell proliferation, migration and wound healing, which was also observed in the histological examinations as part of the *in vivo* study. Second, the cell cycle, mitochondrial number/ATP biogenesis and mitochondrial ETC complexes were also increased in ADMSCs after Nanog gene overexpression. Third, LV function was strongly preserved in AMI animals after receiving Nanog^OE^-ADMSCs. Fourth, the results of the present study revealed that the coordinated axis of the Nanog gene upregulated cell survival and proliferation signaling (Figure 10), resulting in LV tissue regeneration along with a reduction in LV remodeling and an improvement in heart function.

Numerous studies have shown that cell therapies, especially those involving MSCs and ADMSCs, significantly improve ischemia-related LV dysfunction 7, 10, 22, 25-28. The most important finding in the present study was that, compared with AMI animals without treatment, the LVEF was significantly improved in AMI animals after receiving ADMSCs therapy and further significantly improved in those of AMI animals after receiving Nanog^OE^-ADMSCs therapy. Additionally, two imaging parameters, LVRI and LVEDd, and anatomical parameters of the LV chamber size were assessed. These three indices of LV remodeling were significantly lower in the ADMSCs-treated AMI animals and more significantly reduced in the Nanog^OE^-ADMSCs-treated AMI animals than in the untreated AMI animals and the animals treated with siRNA-Nanog in ADMSCs. Furthermore, the pathological findings demonstrated that the infarct area and fibrotic area were identical to the LV chamber size among the groups. Importantly, when the Nanog gene was silenced in ADMSCs, all the abovementioned parameters were markedly reversed in the *in vivo* study. Our findings, in addition to extending the findings of previous studies 7, 10, 22, 25-28, highlight that the Nanog gene, which is involved in tissue regeneration and preservation of heart function, potentially plays a crucial role in the AMI setting. Accordingly, the results of the present study encourage to consider the clinical application of this regimen for AMI patients if the ethical issue is no longer taken into consideration.

Overexpressing and silencing the Nanog gene helped to verify its crucial role in cellular biological effects, leading to the discovery of several noteworthy phenomena. First, Nanog overexpression increased cell proliferation, migration and the wound healing rate. Second, Nanog gene overexpression upregulated the cell cycle and accelerated the rate of mitochondrial ETC complex formation. These findings were observed not only *in vitro* but also *in vivo*. Third, these findings were notably reversed by silencing the Nanog gene in ADMSCs, indicating that manipulation of the Nanog gene is fundamental for regenerative medicine in ischemia-related organ dysfunction.

We next explored the underlying mechanism by which Nanog^OE^-ADMSCs treatment preserves heart function in rodents after AMI. The principal findings of the *in vitro* and *in vivo* studies were that, compared with that in the controls, the expression of JAK/STAT3, a critical transporter of extracellular signals to the intracellular environment, was markedly suppressed in the AMI model. In addition, its expression was markedly increased in the AMI model treated with ADMSCs and markedly increased in the AMI model treated with Nanog^OE^-ADMSCs. Additionally, both cell proliferation (i.e., PI3K/Akt/mTOR) and cell stress signaling (i.e., RAS/RAF/MEK_1/2_/ERK_1/2_) also exhibited similar patterns of JAK/STAT3 signaling among the groups. On the basis of the findings from *in vitro* and *in vivo* studies, we propose that Nanog gene overexpression may activate JAK/STAT3-mediated cell proliferation and cell stress signaling, as schematically illustrated in Figure 10.

The links between LV myocardial damage and oxidative stress/apoptosis have been extensively explored in previous studies 18, 20, 22, 23, 26. Our *in vitro* and *in vivo* studies revealed that, compared with those in H_2_O_2_-treated ADMSCs and AMI animals, oxidative stress and cellular apoptosis were substantially suppressed by Nanog gene overexpression. Our findings, in addition to being consistent with the findings of previous studies 18, 20, 22, 23, 26, partially explain why the LV infarction area was mostly reduced by Nanog^OE^-ADMSCs treatment.

### Study limitations

Our study has several limitations. First, although the results of this study are promising, the study period was relatively short (i.e., only 35 days from AMI induction to the end of the study period). Accordingly, the long-term impact of Nanog^OE^-ADMSCs therapy on improving LV function remains uncertain. Second, although JAK/STAT3-mediated cell survival and cell stress signaling pathways were identified as important for the recovery of heart function after AMI, it remains unclear whether these are the only signaling pathways activated by the Nanog gene after AMI.

In conclusion, Nanog^OE^-ADMSCs therapy activates the JAK/STAT3 pathway-mediated upregulation of cell proliferation and cell stress signaling, which further activates the expression of downstream proteins related to the cell cycle and mitochondrial ETC complexes to improve heart function in AMI rats.

## Figures and Tables

**Figure 2:**
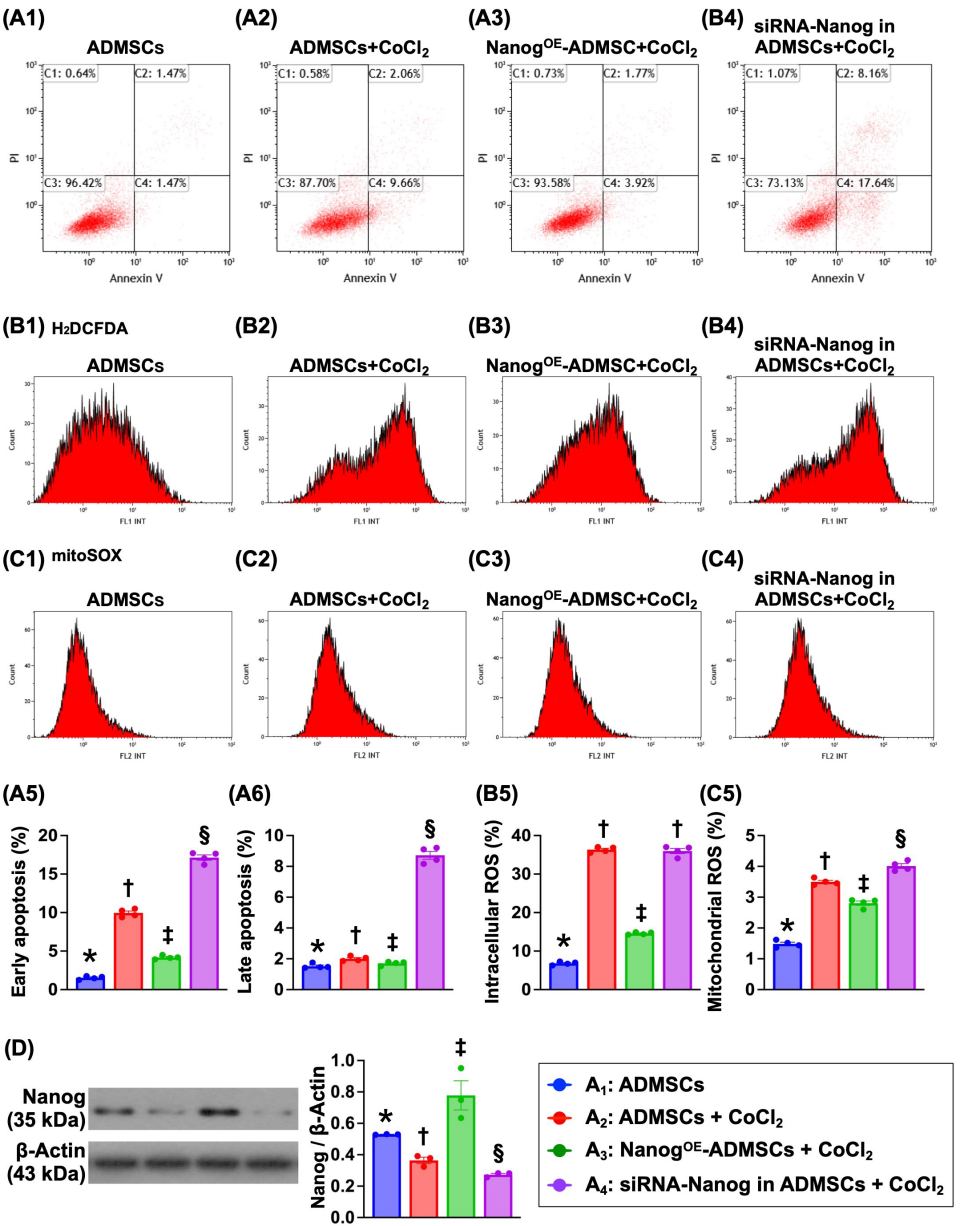
** Impact of Nanog^OE^ in ADMSCs ensured cell survival in hypoxia environments. A1 to A4)** Illustrating the flow cytometric analysis for identification of early (V+/PI-) and late (V+/PI+) apoptosis. **A5)** Analytical result of early apoptosis, * vs. other groups with different symbols (†, ‡, §), *P<*0.0001. **A6)** Analytical result of late apoptosis, * vs. other groups with different symbols (†, ‡, §), *P<*0.0001. **B1 to B4)** Illustrating the flow cytometric analysis for identification of total intracellualr reactive oxygen species (ROS) (i.e., by H_2_DCFDA dye stain). **B5)** Analytical result of the total intracellular ROS, * vs. other groups with different symbols (†, ‡), *P<*0.001. **C1 to C4)** Showing the flow cytometric analysis for identification of mitochondrial ROS (by mitoSOX dye stain). **C5)** Analytical result of mitochondrial ROS by 72h, * vs. other groups with different symbols (†, ‡), *P<*0.001. Note: * indicated the control group. D) Protein level of Nanog, * vs. other groups with different symbols (†, ‡, §), *P<*0.001. All statistical analyses were performed by one-way ANOVA, followed by Bonferroni multiple comparison post hoc test (n=6) for each group. Symbols (*, †, ‡, §) indicate significance (at 0.05 level). Cell grouping: A_1_ = ADMSCs, A_2_ = ADMSCs + CoCl_2_, A_3_ = Nanog^OE^-ADMSCs + CoCl_2_, A4 = siRNA-Nanog in ADMSCs + CoCl_2_. ADMSCs = adipose-derived mesenchymal stem cells; Nanog^OE^ = overexpression of Nanog gene.

**Figure 3:**
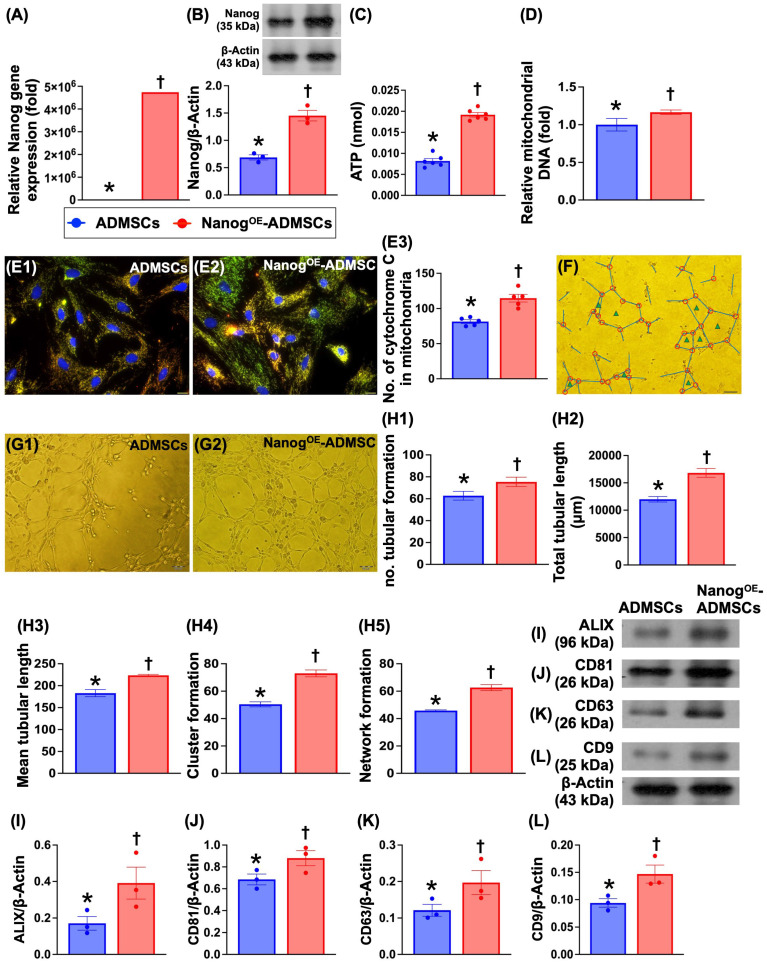
** ATP concentration, relative mitochondria DNA expression, mitochondrial cytochrome-C, and angiogenesis were upregulated by Nanog gene overexpression. A)** Relative gene expression of Nanog in ADMSCs with and without Nanog overexpression, * vs. †, *P<*0.001. **B)** Protein expression of Nanog in ADMSCs with and without Nanog overexpression, * vs. †, *P<*0.001. **C)** ATP concentration in ADMSCs with and without Nanog overexpression, * vs. †, *P<*0.001. **D)** Relative mitochondrial DNA expression, * vs. †, *P<*0.0001. **E1** and **E2)** Illustrating the immunofluorescent (IF) microscopic finding (400x) for identification of mitochondrial cytochrome C (yellow-green color of a merge picture of double stains including red-color Hsp60 stain and green color cytochrome C stain). The scale bars in right lower corner represent 20 µm. **E3)** Analytical result of number of mitochondrial cytochrome C in ADMSCs with and without Nanog overexpression, * vs. †, *P <* 0.0001. **F)** Illustrating the interpretation of Matrigel assay for identification of angiogenesis, including tubular formation (blue length), cluster formation (red circle) network formation (green triangle). **G1** and **G2)** Illustrating one sample of Matrigel assay for identification of rat circulatory derived endothelial progenitor cells (EPCs)-formed angiogenesis in ADMSCs-derived medium treated EPCs (G1) and Nanog^OE^-ADMSCs-derived medium treated EPCs (G2). **H1)** Number of tubular formation, * vs. †, *P <* 0.001. **H2)** Total tubular length, * vs. †, *P<*0.001. **H3)** Mean tubular length, * vs. †, *P<*0.001. **H4)** Cluster formation, * vs. †, *P<*0.001. **H5)** Network formation. **I)** Protein expression of Alix, * vs. †, *P<*0.001. **J)** Protein expression of CD81, * vs. †, *P <* 0.001. **K)** Protein expression of CD63, * vs. †, *P<*0.001. **L)** Protein expression of CD9, * vs. †, *P<*0.001. Note the typical specific biomarkers, including ALIX, CD81, CD63 and C9 were identified from those of ADMSCs- or Nanog^OE^-ADMSCs-derived exosomes. All statistical analyses were performed by one-way ANOVA, followed by Bonferroni multiple comparison post hoc test (n = 3-6) for each group. Symbols (*, †, ‡, §) indicate significance (at 0.05 level). ADMSCs = adipose-derived mesenchymal stem cells; Nanog^OE^ = overexpression of Nanog gene.

**Figure 4:**
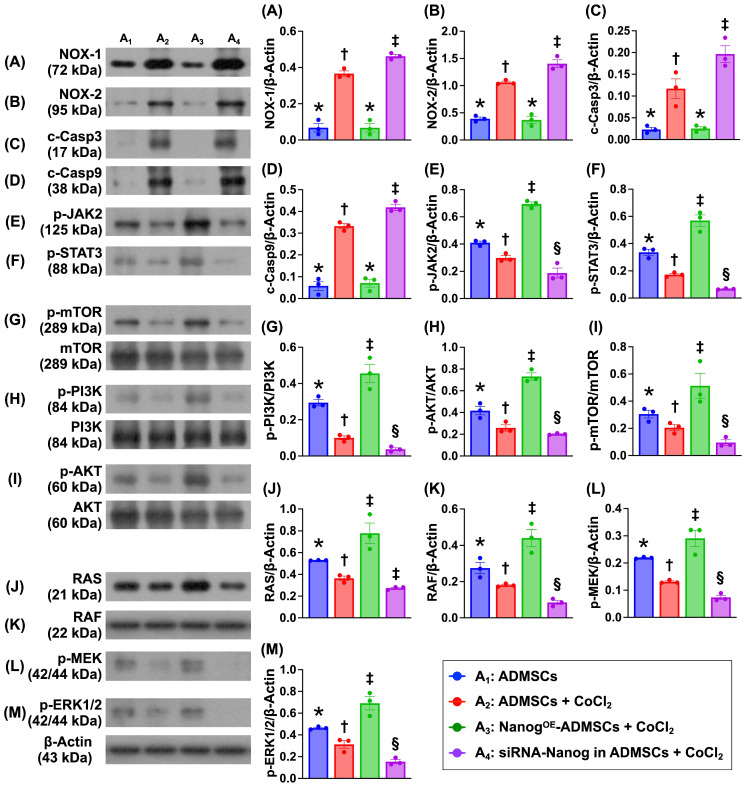
** Nanog gene overexpression in ADMSCs suppressed oxidative stress and cellular apoptosis and upregulated cell stress signaling. A)** Protein expression of NOX-1, * vs. other groups with different symbols (†, ‡), *P <* 0.001. **B)** Protein expression of NOX-2, Analytical result of wound healing ability, * vs. other groups with different symbols (†, ‡), *P <* 0.001. **C)** Protein expression of cleaved caspase 3 (c-Casp3), * vs. other groups with different symbols (†, ‡), *P <* 0.001. **D)** Protein expression of c-Casp9, * vs. other groups with different symbols (†, ‡), *P<*0.0001. **E)** Protein expression of phosphorylated (p)-JAK2, * vs. other groups with different symbols (†, ‡, §), *P<*0.001. **F)** Protein expression of p-STAT, * vs. other groups with different symbols (†, ‡, §), *P<*0.001. **G)** Protein expression of p-m-TOR, * vs. other groups with different symbols (†, ‡, §), *P <* 0.001. H) Protein expression of p-PI3K, * vs. other groups with different symbols (†, ‡, §), *P <* 0.0001. I) Protein expression of p-Akt, * vs. other groups with different symbols (†, ‡, §), *P <* 0.001. J) Protein expression of Ras, * vs. other groups with different symbols (†, ‡, §), *P<*0.001. K) Protein expression of Raf, * vs. other groups with different symbols (†, ‡, §), *P<*0.0001. L) Protein expression of p-MEK1/2, * vs. other groups with different symbols (†, ‡, §), *P<*0.001. M) Protein expression of p-ERK1/2, * vs. other groups with different symbols (†, ‡, §), *P <* 0.001. Note: * indicated the control group. All statistical analyses were performed by one-way ANOVA, followed by Bonferroni multiple comparison post hoc test (n = 3) for each group. Symbols (*, †, ‡, §) indicate significance (at 0.05 level). Cell grouping: A_1_ = ADMSCs, A_2_ = ADMSCs + CoCl_2_, A_3_ = Nanog^OE^-ADMSCs + CoCl_2_, A4 = siRNA-Nanog in ADMSCs + CoCl_2_. ADMSCs = adipose-derived mesenchymal stem cells; Nanog^OE^ = overexpression of Nanog gene.

**Figure 5:**
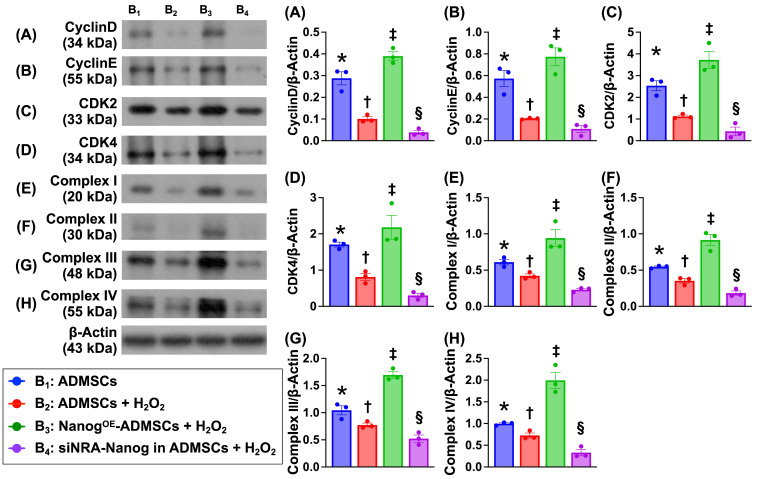
** Nanog gene expression activated cell cycle and upregulated mitochondrial electron transport chain (ETC) in oxidative stress condition. A)** Protein expression of Cyclin D, * vs. other groups with different symbols (†, ‡, §), *P <* 0.0001. **B)** Protein expression of Cyclin E, * vs. other groups with different symbols (†, ‡, §), *P <* 0.001. **C)** Protein expression of CKD2, * vs. other groups with different symbols (†, ‡, §), *P <* 0.0001. **D)** Protein expression of CKD4, * vs. other groups with different symbols (†, ‡, §), *P<*0.0001. **E)** Protein expression of complex I, * vs. other groups with different symbols (†, ‡, §), *P <* 0.001. **F)** Protein expression of complex II, * vs. other groups with different symbols (†, ‡, §), *P <* 0.001. **G)** Protein expression of complex III, * vs. other groups with different symbols (†, ‡, §), *P <* 0.001. **H)** Protein expression of complex IV, * vs. other groups with different symbols (†, ‡, §), *P <* 0.0001. Note: * indicated the control group. All statistical analyses were performed by one-way ANOVA, followed by Bonferroni multiple comparison post hoc test (n = 6) for each group. Symbols (*, †, ‡, §) indicate significance (at 0.05 level). Cell grouping: B_1_ = ADMSCs, B_2_ = ADMSCs + H_2_O_2_, B_3_ = Nanog^OE^-ADMSCs + H_2_O_2_, B_4_ = siRNA-Nanog gene in ADMSCs + H_2_O_2_. ADMSCs = adipose-derived mesenchymal stem cells; Nanog^OE^ = overexpression of Nanog gene.

**Figure 6:**
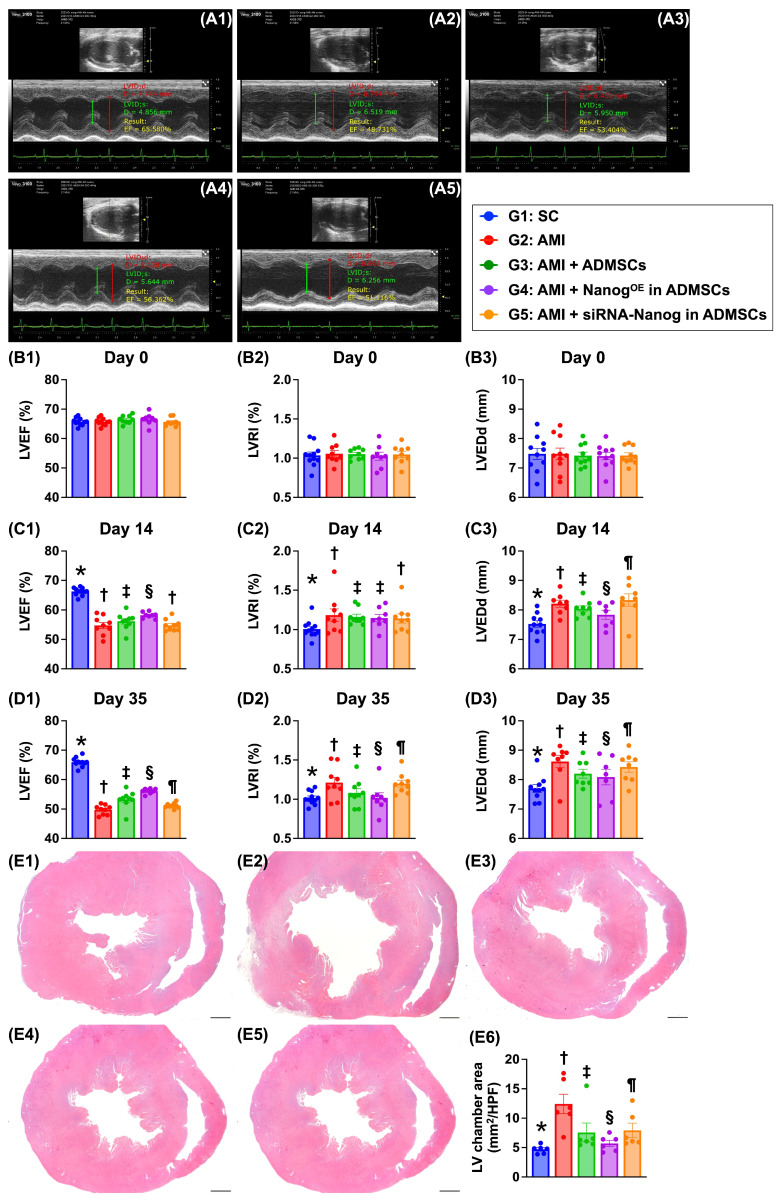
** Time courses of LVEF, LVRI and LVEDd, and LV chamber area by day 35. A1 to A5)** Illustrating the M-mode findings of transthoracic echocardiographic study at the time point of day 35 after AMI induction in each group of the animal. The left ventricular end diastolic dimension (LVEDd) was notably increased in groups 2 and 5 than in other groups by day 35 after AMI induction. **B1)** left ventricular ejection fraction (LVEF) at baseline, *P* > 0.5. **B2)** LVRI at baseline, *P* > 0.5. B3) LVEDd at baseline, *P* > 0.5. **C1)** LVEF at day 14 after AMI induction, * vs. other groups with different symbols (†, ‡, §), *P <* 0.0001. **C2)** LVRI at day 14 after AMI induction, * vs. other groups with different symbols (†, ‡), *P <* 0.0001. **C3)** LVEDd at day 14 after AMI induction, * vs. other groups with different symbols (†, ‡, §, ¶), *P <* 0.0001. **D1)** LVEF at day 35 after AMI induction, * vs. other groups with different symbols (†, ‡, §, ¶), *P <* 0.0001. **D2)** LVRI at day 35 after AMI induction, * vs. other groups with different symbols (†, ‡, §, ¶), *P <* 0.0001. **D3)** LVEDd at day 35 after AMI induction, * vs. other groups with different symbols (†, ‡, §, ¶), *P <* 0.0001. **E1 to E5)** Illustrating the vertical microscopic (cross sections) finding (12.5x) of H.E., stain for identification of LV chamber area. All scale bars in right lower corner represents 12.5 mm. **E6)** Analytical result of LV chamber area, * vs. other groups with different symbols (†, ‡, §, ¶), *P <* 0.0001. Note: * indicated the control group. All statistical analyses were performed by one-way ANOVA, followed by Bonferroni multiple comparison post hoc test (n = 8-10) for each group. Symbols (*, †, ‡, §, ¶) indicate significance (at 0.05 level). Group 1 = sham-operated control (SC), group 2 = AMI, group 3 = AMI + ADMSCs, group 4 = AMI + Nanog^OE^ in ADMSCs, 5 = AMI + siRNA-Nanog in ADMSCs. LV = left ventricular; AMI = acute myocardial infarction; ADMSCs = adipose-derived mesenchymal stem cells; Nanog^OE^ = overexpression of Nanog gene.

**Figure 7:**
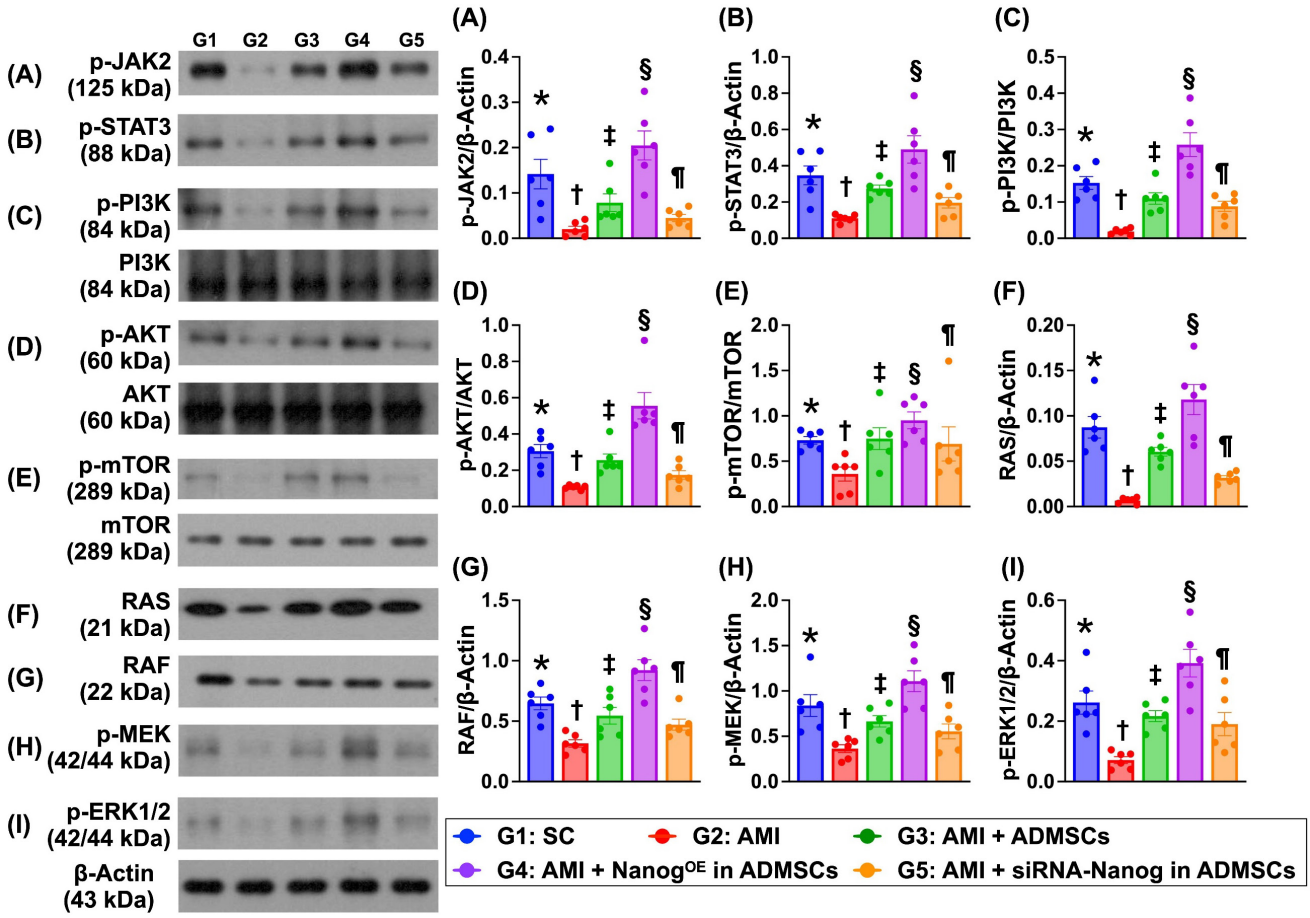
** Protein levels of cell proliferation and cell-stress signalings in LV myocardium by day 35 after AMI induction. A)** Protein expression of phosphorylated (p)-JAK, * vs. other groups with different symbols (†, ‡, §, ¶), *P<*0.0001. **B)** Protein expression of p-STAT3, * vs. other groups with different symbols (†, ‡, §, ¶), *P<*0.0001. **C)** Protein expression of p-PI3K, * vs. other groups with different symbols (†, ‡, §, ¶), *P<*0.0001. **D)** Protein expression of p-Akt, * vs. other groups with different symbols (†, ‡, §, ¶), *P<*0.0001. **E)** Protein expression of p-mTOR, * vs. other groups with different symbols (†, ‡, §, ¶), *P<*0.0001. **F)** Protein expression of RAS, * vs. other groups with different symbols (†, ‡, §, ¶), *P<*0.0001. **G)** Protein expression of RAF, * vs. other groups with different symbols (†, ‡, §, ¶), *P<*0.0001. **H)** Protein expression of p-MEK1/2, * vs. other groups with different symbols (†, ‡, §, ¶), *P<*0.0001. **I)** Protein expression of ERK1/2, * vs. other groups with different symbols (†, ‡, §, ¶), *P<*0.0001. Note: * indicated the control group. All statistical analyses were performed by one-way ANOVA, followed by Bonferroni multiple comparison post hoc test (n=6) for each group. Symbols (*, †, ‡, §, ¶) indicate significance (at 0.05 level). LV = left ventricular; SC = sham-operated control; AMI = acute myocardial infarction; ADMSCs = adipose-derived mesenchymal stem cells; Nanog^OE^ = overexpression of Nanog gene.

**Figure 8:**
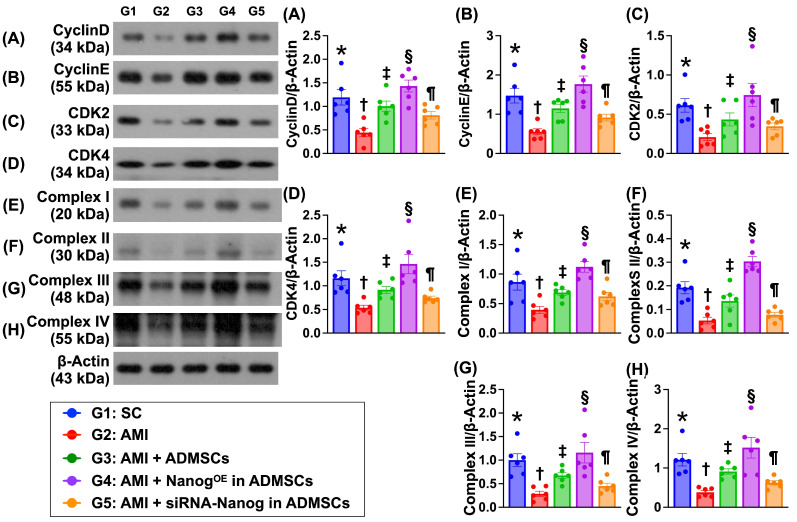
** Protein expressions of cell cycle and mitochondrial ETC complexes in LV myocardium by day 35 after AMI induction. A)** Protein expression of cyclin D, * vs. other groups with different symbols (†, ‡, §, ¶), *P <* 0.0001. **B)** Protein expression of cyclin E, * vs. other groups with different symbols (†, ‡, §, ¶), *P <* 0.0001. **C)** Protein expression of CDK2, * vs. other groups with different symbols (†, ‡, §, ¶), *P <* 0.0001. **D)** Protein expression of CDK4, * vs. other groups with different symbols (†, ‡, §, ¶), *P <* 0.0001. **E)** Protein expression of complex I, * vs. other groups with different symbols (†, ‡, §, ¶), *P <* 0.0001. **F)** Protein expression of complex II, * vs. other groups with different symbols (†, ‡, §, ¶), *P <* 0.0001. **G)** Protein expression of complex III, * vs. other groups with different symbols (†, ‡, §, ¶), *P <* 0.0001. **H)** Protein expression of complex IV, * vs. other groups with different symbols (†, ‡, §, ¶), *P <* 0.0001. Note: * indicated the control group. All statistical analyses were performed by one-way ANOVA, followed by Bonferroni multiple comparison post hoc test (n = 6) for each group. Symbols (*, †, ‡, §, ¶) indicate significance (at 0.05 level). LV = left ventricular; SC = sham-operated control; AMI = acute myocardial infarction; ADMSCs = adipose-derived mesenchymal stem cells; Nanog^OE^ = overexpression of Nanog gene.

**Figure 9:**
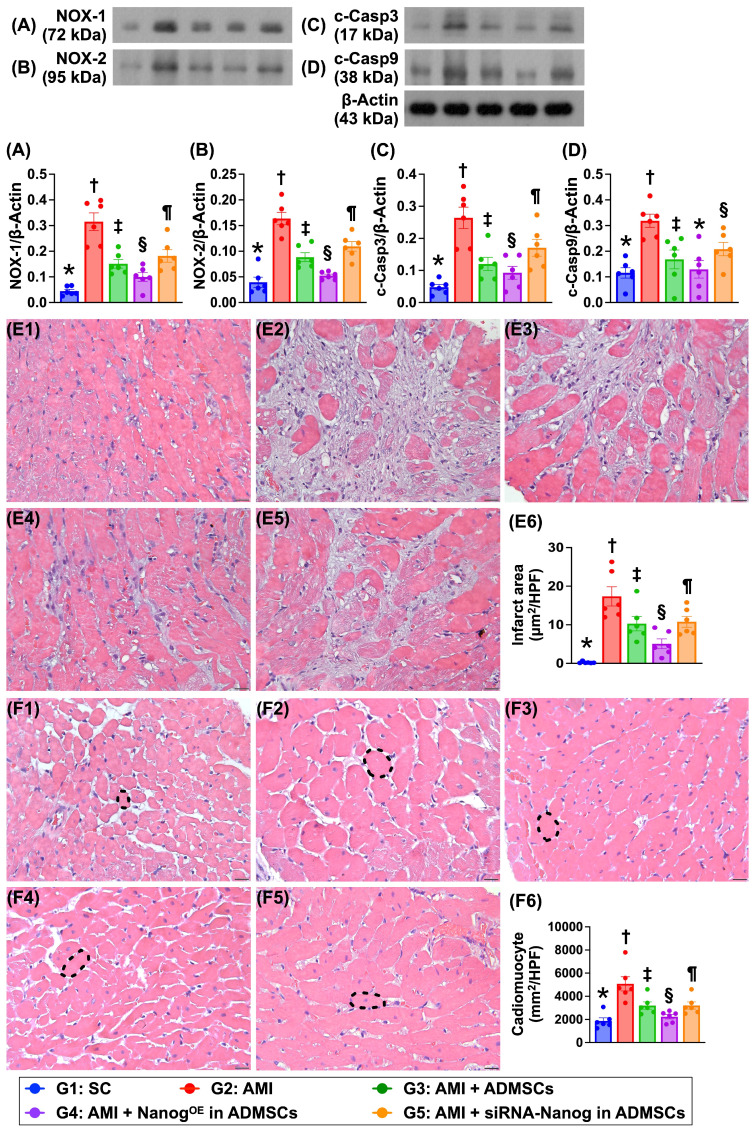
**Protein expressions of oxidative stress and apoptosis, cardiomyocyte size and LV myocardial infarct area. A)** Protein expression of NOX-1, * vs. other groups with different symbols (†, ‡, §, ¶), *P <* 0.0001. **B)** Protein expression of NOX-2, * vs. other groups with different symbols (†, ‡, §, ¶), *P <* 0.0001. **C)** Protein expression of cleaved caspase 3 (c-Casp3, * vs. other groups with different symbols (†, ‡, §, ¶), *P <* 0.0001. **D)** Protein expression of c-Casp9, * vs. other groups with different symbols (†, ‡, §, ¶), *P <* 0.0001. **E1 to E5)** Illustrating the microscopic finding (200x) of H.E., stain for identification of infarct area of LV myocardium (white color). All scale bar in right lower corner represents 50 µm. **E6)** Analytical result of infarct area, * vs. other groups with different symbols (†, ‡, §, ¶), *P <* 0.0001. **F1 to F5)** Showing the microscopic finding (400x) of H.E., stain for identification of cardiomyocyte size (black dotted line) of LV myocardium. **F6)** Analytical result of cardiomyocyte size, * vs. other groups with different symbols (†, ‡, §, ¶), *P <* 0.0001. All scale bar in right lower corner represents 20.0 µm. Note: * indicated the control group. All statistical analyses were performed by one-way ANOVA, followed by Bonferroni multiple comparison post hoc test (n = 6) for each group. Symbols (*, †, ‡, §, ¶) indicate significance (at 0.05 level). LV = left ventricular; SC = sham-operated control; AMI = acute myocardial infarction; ADMSCs = adipose-derived mesenchymal stem cells; Nanog^OE^ = overexpression of Nanog gene.

## Abbreviations

Nanog^OE^-ADMSCs: *NANOG* gene overexpression on adipose-derived mesenchymal stem cells
siRNA-Nanog-ADMSCs: small interfering RNA (i.e., silencing) *Nanog* gene in ADMSCs
AMI: acute myocardial infarction
MTT: (3-(4,5-Dimethylthiazol-2-yl)-2,5-diphenyltetrazolium bromide)
CoCl_2_: Cobalt chloride
MSCs: mesenchymal stem cells
LV: left ventricle
ROS: reactive oxygen species
LVEF: left ventricular ejection fraction
HF: heart failure
LCA: left coronary artery
LVESd: left ventricular end-systolic diameter
LVEDd: left ventricular end-diastolic diameter
IHC: immunohistochemical
ETC: electron transport chain
LVRI: left ventricular remodeling index
